# Research on the Application of Diamond Film in Chemical Mechanical Polishing

**DOI:** 10.3390/nano16050332

**Published:** 2026-03-06

**Authors:** Yibao Wang, Xueyu Zhang, Fengxiang Guo, Mei Zhang, Xiaoling Sun, Lili Zhang, Guangsen Xia, Xu Chai, Shaoyan Wang, Xuesong Zhou, Zhigang Gai

**Affiliations:** State Key Laboratory of Physical Oceanography, Institute of Oceanographic Instrumentation, Qilu University of Technology, Shandong Academy of Sciences, Qingdao 266000, China; yibaowang2021@163.com (Y.W.);

**Keywords:** diamond films, CMP, friction

## Abstract

Polishing pad conditioners are of critical importance in chemical mechanical polishing (CMP), acting as a key determinant of CMP efficiency and an indispensable consumable in the polishing process. In addition to acid–alkali resistance and outstanding stability, stringent requirements are also imposed on the physical properties of conditioners, including high hardness and wear resistance. Diamond films, with their exceptional comprehensive performance, can satisfactorily fulfill these demanding specifications. In this work, to investigate the bonding strength and wear resistance of diamond films deposited on a silicon carbide (SiC) substrate, four groups of diamond films with distinct processing parameters were synthesized via hot wire chemical vapor deposition (HWCVD) on SiC substrates. Nano-scratch tests were employed to characterize the bonding strength at the diamond film/SiC substrate interface, while wear tests under humid conditions with a 500 g load, accompanied by in-depth analysis of the associated wear mechanisms, were conducted. The results demonstrate that diamond films exhibit tremendous application potential as CMP pad conditioners in CMP processes.

## 1. Introduction

Chemical mechanical polishing (CMP), as the core technology for achieving global planarization in the semiconductor manufacturing field, has witnessed a simultaneous increase in process complexity and the number of process steps with the continuous evolution of chip manufacturing processes toward smaller nodes, so as to meet the increasingly stringent flatness requirements of device surfaces [1]. As a key functional component in the polishing system, the stability of the surface pore structure of the CMP pad directly determines the polishing efficiency and the surface quality of workpieces. Trimmers loaded with irregular diamond particles on their surfaces can effectively restore the original porosity of the polishing pad surface and maintain its polishing performance [2,3,4,5].

With the continuous shrinkage of chip manufacturing process nodes, the control standards for metal contaminants in devices have become increasingly strict. Chemical vapor deposition (CVD)-type diamond trimmers are gradually replacing traditional trimmers due to their excellent wear resistance, chemical stability, and low-metal-contamination characteristics, becoming an important direction for industrial technological upgrading. A number of traditional trimmer manufacturers have also launched research, development and industrialization of such products.

At present, most studies on CMP trimmers focus on anti-metal-contamination modification, substrate material selection, pyramid-shaped diamond abrasive spacing [6], laser parameter optimization [7,8,9], and precision processing technology of ceramic substrates [10,11,12]. However, systematic in-depth analysis has not been carried out on key scientific issues such as the precise regulation of diamond film roughness, the quantitative characterization of film–substrate bonding strength, and the friction and wear mechanism of diamond films under CMP conditions, resulting in obvious shortcomings in related research.

With the aim of optimizing the performance of diamond trimmers for CMP, this study prepared diamond films with gradient roughness by CVD technology. Scanning electron microscopy (SEM), Raman spectroscopy, and atomic force microscopy (AFM) were used to characterize and analyze the films, while a nanoindenter was employed to quantitatively test the bonding strength between the carbide substrate and the diamond film. Under wettability conditions simulating the actual operating environment, friction and wear experiments and wear mechanism analysis were carried out to screen out the optimal diamond film suitable for the CMP field. This work lays a foundation for the application of diamond in this field and further explores its application potential.

## 2. Materials and Methods

### 2.1. Preparation of Diamond Film

After patterning the SiC substrate (PUZE, Qingdao, China), the diamond film with a thickness of 10 μm was deposited on the substrate by HFCVD (Institute of Oceanographic Instrumentation, Qingdao, China).

**Figure 1:**
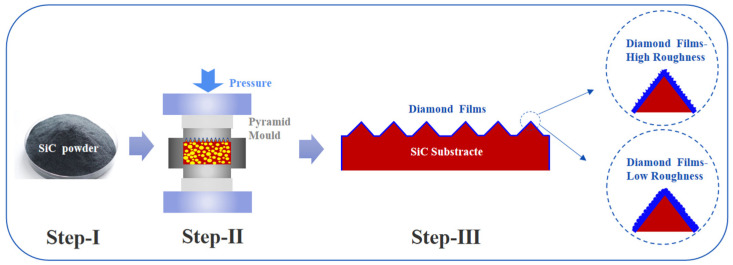
Processing schematic diagram of new-type pad conditioner.

Four different parameters of diamond coatings were prepared, with the variable being the control of the grain size of the diamond surface, as shown in Figure 1, Step III, depositing diamond films of different roughnesses on the protruding surface of the pyramid. As shown in Table 1, at a chamber pressure of 5 kPa and a substrate temperature of 850 °C, diamond films with roughness (Sa) ranging from 61 to 125 nm were obtained by adjusting the CH_4_/H_2_ ratio in order to verify which grain size is more suitable for the dresser. Their numbers are 1#, 2#, 3# and 4#. SEM (Regulus 8100, Hitachi High-Tech, Tokyo, Japan), Raman spectroscopy (LabRAM, HORIBA Scientific, Palaiseau, France, 532 nm), and AFM (AMF500II, Hitachi High-Tech, Tokyo, Japan) confirmed that the film was made of high-quality diamond.

### 2.2. Combined Strength Test

The critical load, initial load and final load of the coating were quantitatively characterized using a nano-scratch instrument. The initial load and final load were 0 and 100 N, respectively. During the test, the scratch length was 3 mm, the loading time was 1 min, and the loading rate was 100 N/min. During the test, the friction force signal was collected. The critical load of the coating was determined by the loading force corresponding to the drastic change in friction force. A Rockwell hardness tester was adopted for indentation tests under a load of 100 kgf, and the film peeling state around the indentation was observed and analyzed to quantitatively evaluate the bonding strength between the carbide substrate and the diamond film.

### 2.3. Friction–Wear Test

The application conditions for the CMP finishing module are a pressure of 4.5 kg/cm^2^ and a rotational speed of 90 rpm. To verify the stability and durability of the film in a dynamic environment, a zirconia ball with a diameter of 3 mm was used as the friction pair, with a ball cross-sectional area of 0.07065 cm^2^. Therefore, a load of 317 g can meet these requirements. Friction and wear parameters: 3 mm diameter zirconia ball, original weight of the ball 0.0863 g, applied load 500 g, tests conducted under wet conditions respectively, disk rotational speed 90 rpm, and test time 60 min.

## 3. Results

**Figure 2:**
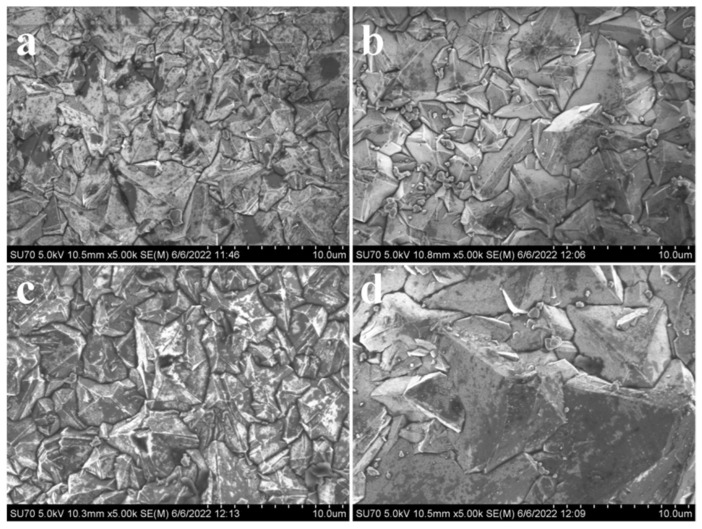
SEM of diamond films under different parameters: (**a**): Sample 1#; (**b**): Sample 2#; (**c**): Sample 3#; (**d**): Sample 4#.

Figure 2 shows the scanning electron microscope (SEM) images of different samples. By observing these images, it can be found that as the methane concentration decreases, the grain size of the film significantly increases. When the methane concentration is low, the concentration of active H atoms is high, which has a good etching effect on sp^2^ graphite carbon, easily causing the CH_3_-methyl group to dehydrogenate and form a diamond structure with sp^3^ bonds [13]. As a result, the grain facets are clear and the edges are distinct. The morphology of the diamond film with high methane concentration shows a relatively smooth surface (Figure 2a), while when the concentration decreases, the surface smoothness also decreases (Figure 2b). The roughness of the observed patterns varies with different methane concentrations. The higher the methane concentration, the lower the roughness. When the methane concentration decreases from 2.5% to 1.5%, the grain sizes of Samples 1#, 2#, and 3# change slightly, to approximately 5 µm. When the methane concentration drops to 1%, the grain size of Sample 4# significantly increases, reaching approximately 10 µm.

**Figure 3:**
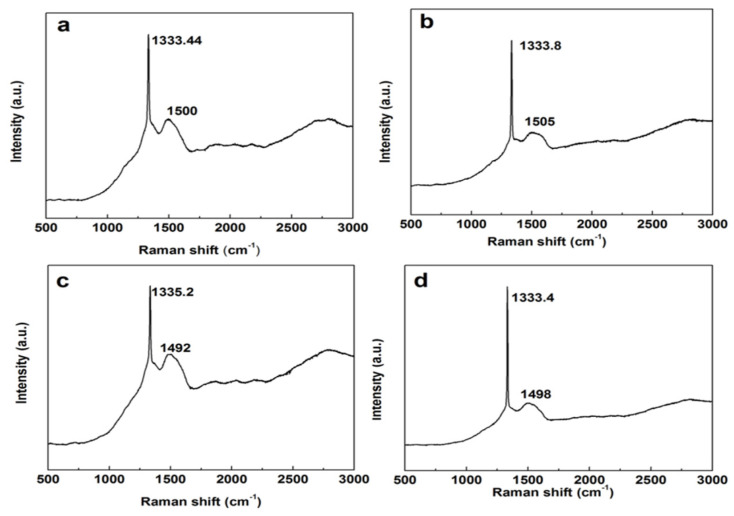
Raman of diamond films: (**a**): Sample 1#; (**b**): Sample 2#; (**c**): Sample 3#; (**d**): Sample 4#.

Figure 3 presents the Raman spectra of different samples. There are mainly three forms of carbon atoms on the diamond surface. The first form consists of a diamond structure with sp^3^ carbon bonds, manifested as a characteristic peak around 1332 cm^−1^. A broad peak envelope observed in the range of 1450–1550 cm^−1^ is attributed to the vibrational modes of disordered carbon at grain boundaries, which indicates the presence of disordered carbon in the sample. Meanwhile, a distinct peak envelope at 2700 cm^−1^ demonstrates the existence of a small amount of graphite [14,15,16]. From Figure 3, it can be observed that the Raman characteristic peak of the diamond film shifts towards a higher wavenumber. This is primarily due to the difference in thermal expansion coefficients between diamond and the silicon carbide substrate, resulting in compressive stress on the diamond film at room temperature. The magnitude of the residual stress can be determined according to the formula σ = −0.567 (ν − ν_0_) GPa/cm^−1^, where ν_0_ = 1332 cm^−1^ and ν is the measured Raman shift of diamonds with different coating structures [17]. Therefore, the compressive stresses experienced by Samples 1#–4# are −0.816 GPa, −1.02 GPa, −1.81 GPa, and −0.79 GPa, respectively. Compared with other substrates such as tungsten carbide, tantalum, and niobium, the thermal stress is approximately half as small, indicating that diamond has more compatible lattice matching with silicon carbide.

**Figure 4:**
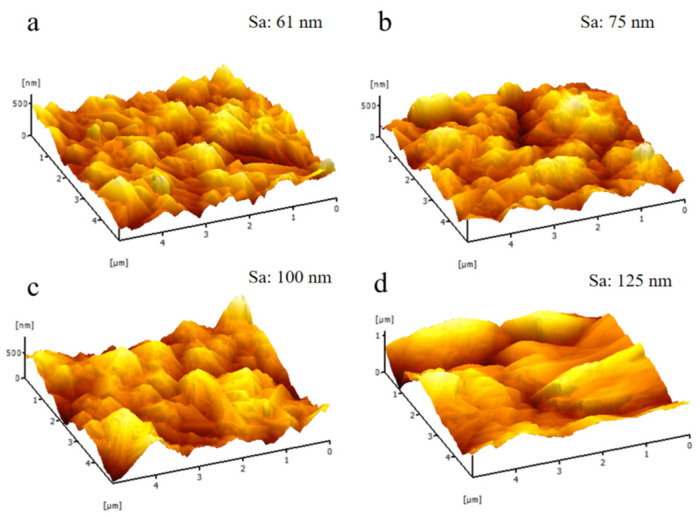
Surface roughness (5 µm × 5 µm): (**a**): Sample 1#; (**b**): Sample 2#; (**c**): Sample 3#; (**d**): Sample 4#.

Using atomic force microscopy (AFM), the surface topography of four diamond films with varying roughness levels was quantitatively characterized. The areal roughness parameter Sa was determined to be 61 nm (Sample 1#), 75 nm (Sample 2#), 100 nm (Sample 3#), and 125 nm (Sample 4#), as illustrated in Figure 4. The results indicate a clear trend of increasing surface roughness with decreasing methane concentration during deposition. This correlation is consistent with prior scanning electron microscopy (SEM) observations.

**Figure 5:**
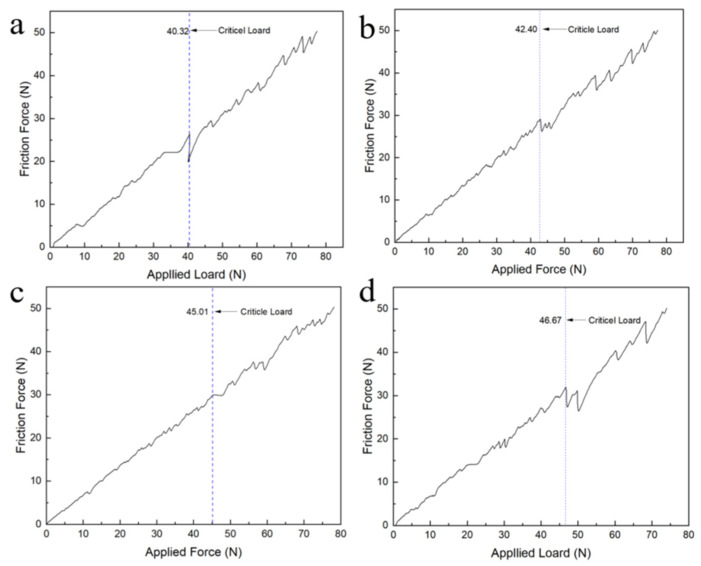
Scratch tests were performed on four samples, with friction and loading force curves: (**a**): Sample 1#; (**b**): Sample 2#; (**c**): Sample 3#; (**d**): Sample 4#.

The Rockwell scratch tester is employed to quantitatively characterize the critical load of the coating. The initial load and the final load are set at 0 and 100 N, respectively. During the testing process, the scratch length is 3 mm, the loading time is 1 min, and the loading rate is 100 N/min [18,19]. The friction force signal is collected during the test, and the critical load of the coating is determined by the corresponding loading force when the friction force changes abruptly. As illustrated in Figure 5, the measured critical loads for Samples #1 through #4 are 40.32 N, 42.40 N, 45.01 N, and 46.67 N, respectively. The consistently high adhesion strength observed across all specimens can be attributed to the favorable formation of Si–C covalent bonds between the diamond films and the silicon carbide substrate [20,21]. Furthermore, a positive correlation is noted between the critical load and the surface grain size of the films. This trend is likely due to the predominance of sp^3^-hybridized carbon structures promoted under lower carbon-source conditions during the initial nucleation stage of diamond growth, which effectively suppresses sp^2^ formation and enhances covalent bonding at the film–substrate interface.

**Figure 6:**
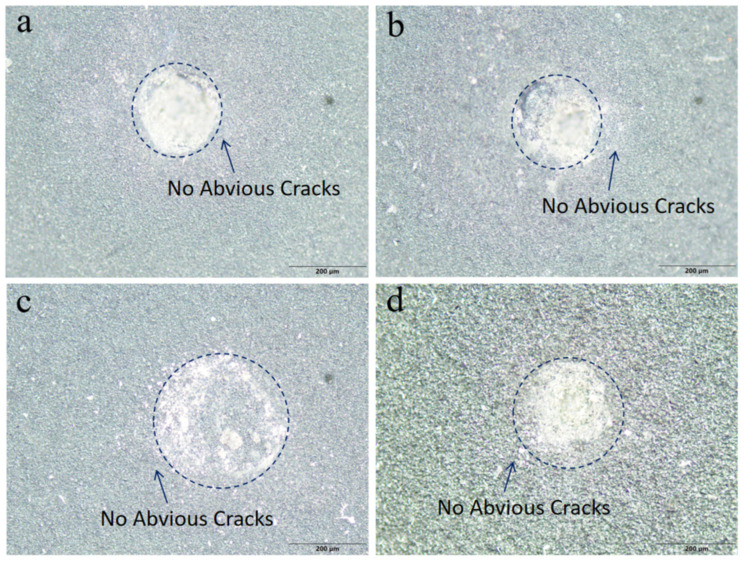
Rockwell indentation test on four samples with 100 kgf: (**a**): Sample 1#; (**b**): Sample 2#; (**c**): Sample 3#; (**d**): Sample 4#.

To intuitively evaluate the bonding strength between the diamond film and the silicon carbide substrate, the Rockwell indentation test was performed under a load of 100 kgf, with the crack morphology around the indentation observed and analyzed, as shown in Figure 6. No cracks or extended delamination areas of the film were observed around the indentations of all samples under the applied load of 100 kgf. Consistent with the results of the scratch test, a robust interface was formed between the diamond film and the silicon carbide substrate, which lays a solid foundation for subsequent friction and wear tests.

Based on the above analyses, we obtained four types of diamond film samples with progressively increasing roughness, which exhibit high bonding strength. To further investigate the friction and wear properties corresponding to different roughness levels, systematic analyses were conducted on the friction coefficient and wear rate of zirconia grinding balls.

**Figure 7:**
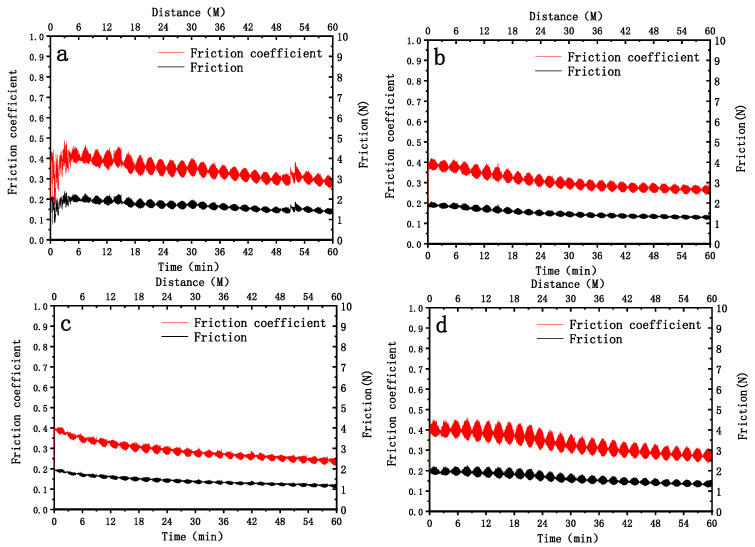
Friction coefficient curves of four samples under 500 g load under water wetting conditions: (**a**): Sample 1#; (**b**): Sample 2#; (**c**): Sample 3#; (**d**): Sample 4#.

Figure 7 shows the friction coefficient and friction force variation curves of four groups of samples under a constant underwater load of 500 g. The data reveal significant quantitative differences in the friction responses among the groups, reflecting the heterogeneity of interfacial friction behaviors. According to the graph data, the friction coefficient of Sample 1# rapidly rises to approximately 0.4 within the initial 5 min and then enters a stable fluctuation stage, with the fluctuation range controlled between 0.3 and 0.4. For Sample 2#, the friction coefficient quickly converges to 0.35, and the overall fluctuation amplitude is less than 0.03, exhibiting the optimal interfacial stability among the four groups. Sample 3# presents an obvious decreasing trend in the friction coefficient, gradually dropping from an initial value of 0.4 to a stable value of 0.3 with a decreasing rate of about 0.02 per minute. Sample 4# shows the most intense fluctuation in the friction coefficient, with a range of 0.3 to 0.5 and a difference of 0.2 between the peak and valley values, and there is no obvious stable stage, indicating an extremely unstable interfacial friction state. The underwater wet environment maintains the friction coefficients of all samples within the range of 0.2 to 0.5, which is significantly lower than that in the dry environment (usually >0.5), confirming the fluid lubrication effect of the aqueous medium.

The quantitative differences in friction performance among the groups essentially stem from the variations in material surface properties and underwater interfacial interaction mechanisms, providing key data support for the design of underwater wear-resistant materials. From the perspective of quantitative indicators, taking friction coefficient stability and fluctuation amplitude as the core evaluation criteria, the performance ranking of the four groups of samples is Sample 2# > Sample 1# > Sample 3# > Sample 4#. The low-fluctuation characteristic of Sample 2# is attributed to its excellent underwater wettability and surface homogeneity, which can form a continuous and stable water film lubrication layer to avoid sudden changes in local friction states. The decreasing friction coefficient behavior of Sample 3# corresponds to the underwater interfacial running-in process; after the wear of surface asperities, the contact area is optimized, and the mixed composite lubrication film formed by wear debris and water gradually enhances the friction-reducing effect, with its final stable value of 0.3 demonstrating potential late-stage friction-reducing advantages. The root cause of the intense fluctuation of Sample 4# lies in the heterogeneity of surface wettability, which leads to frequent rupture and reconstruction of the water film, alternating local dry friction and fluid lubrication states, and triggering sudden changes in the friction coefficient. This data suggests that such materials need to improve the uniformity of wettability through surface modification to avoid interfacial failure risks (Table 2).

**Figure 8:**
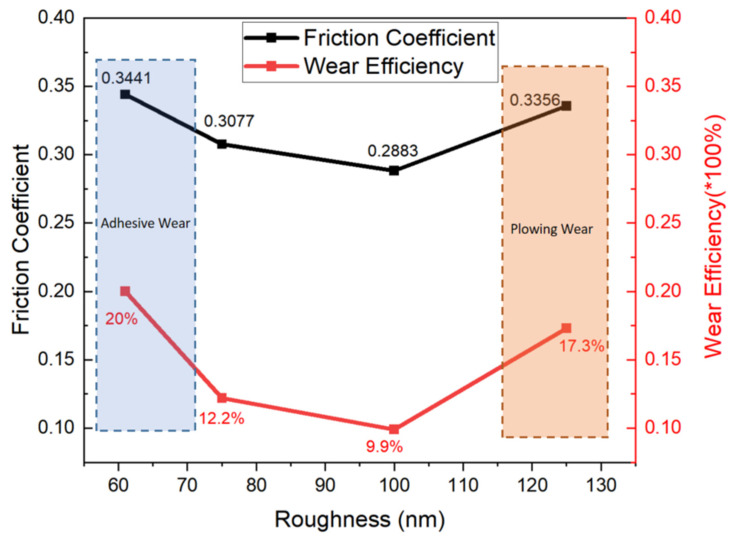
Friction coefficients and wear rates with different surface roughnesses.

In the application scenario of CMP dressers, maximizing the removal rate of zirconia grinding balls is the core demand. As shown in the experimental chart (Figure 8), the diamond film with a roughness of 61 nm exhibits the optimal performance, corresponding to a grinding ball wear efficiency of 20%, which is significantly higher than that of samples with other roughness values—when the roughness increases to 100 nm, the wear efficiency drops to 9.9%, and it further rises to 17.3% as the roughness reaches 125 nm. Notably, this experimental phenomenon deviates from the traditional speculation that “wear efficiency increases with rising roughness”, presenting a distinct non-monotonic variation law. Meanwhile, the friction coefficient data in the chart also shows a completely consistent trend with the wear efficiency: it decreases from 0.3441 at 61 nm to 0.2883 at 100 nm, and then rebounds to 0.3356 at 125 nm, both following a “first decrease and then increase” characteristic.

Analysis of the correlated trends observed in this study offers valuable guidance for the parameter optimization of diamond films for CMP dressers. From the perspective of interface interaction mechanisms, under low roughness (61 nm), the diamond film surface roughness is smaller than the surface undulation scale of the zirconia grinding balls, which likely leads to the largest real contact area between the two interfaces. This is consistent with established theories that enhanced van der Waals forces and mechanical interlocking at the interface can promote adhesive wear. The zirconia grinding balls exhibit fatigue peeling during cyclic adhesion–detachment, which aligns with the highest wear efficiency of 20% observed in our experimental data, as reported in prior studies on similar contact systems.

When roughness increases to 100 nm, discrete micro-protrusions on the diamond surface may lead to a substantial reduction in the real contact area. Concurrently, under wet conditions, it is well-documented that zirconia particles can form a continuous hydrated lubricating layer with water molecules via hydrogen bonding, which would effectively block direct contact between diamond and grinding balls. This is supported by our observation that the friction coefficient reached a minimum of 0.2883, and the wear efficiency was the lowest at 9.9%, consistent with the lubrication behavior reported in the literature.

When roughness further increases to 125 nm, the conical micro-protrusions on the diamond surface are likely to penetrate the hydrated lubricating layer, resulting in intense mechanical interlocking and scraping with the zirconia ball surface. This transition to plowing wear is supported by our data showing a rebound in the friction coefficient to 0.3356 and an increase in wear efficiency to 17.3%, which remains lower than the peak value at 61 nm. This trend aligns with the established understanding of how surface protrusions disrupt lubrication layers in abrasive contact scenarios.

**Figure 9:**
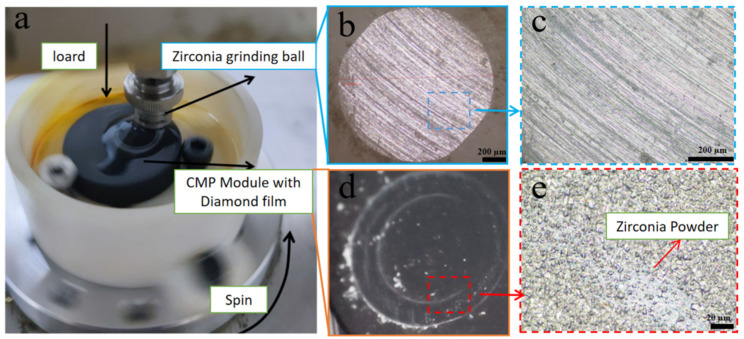
The surfaces of the grinding balls and the diamond after friction and wear. (**a**): Schematic diagram of friction and wear; (**b**): surface of zirconia grinding balls; (**c**): magnified view of the selected area in (**b**); (**d**): surface of diamond; (**e**): magnified view of the selected area in (**d**).

Figure 9 presents the surface morphologies of zirconia grinding balls and diamond films after friction. Figure 9b,c show uniform scratches on the zirconia surface, while Figure 9d,e reveal the accumulation of zirconia wear debris on the diamond film surface. It is precisely these scratches and wear debris accumulation that affect the friction behavior and wear rate.

## 4. Conclusions

In this study, polycrystalline diamond films with a gradient-increasing roughness were fabricated via the hot filament chemical vapor deposition (HFCVD) method, and their application performance in the field of chemical mechanical polishing (CMP) was systematically investigated. The results demonstrate that the thermal stress of the diamond films grown on silicon carbide substrates ranges from −0.8 to −1.8 GPa, which is only 50% of that of the films deposited on metal substrates. The average critical load between the diamond films and silicon carbide substrates exceeds 40 N. Under the working condition of 500 g load (corresponding to a pressure ≥ 7.07 kg/cm^2^), the films exhibited no failure phenomena such as damage or peeling after 1 h of continuous operation, demonstrating excellent structural stability and engineering application potential. Under water-lubricated conditions, the wear mechanisms between the diamond films with different roughnesses and the mating materials exhibit a characteristic evolutionary law of adhesive wear and plowing wear. The conclusions of this study provide an important theoretical basis and experimental support for the performance regulation and structural design of diamond films for CMP dressers.

## Figures and Tables

**Table 1:** Preparation parameters of diamond films with different roughnesses.

Gas Ratio (CH_4_/H_2_, %)	Chamber Pressure	Substrate Temperature	Roughness (Sa, nm)
0.75%	5 kPa	850 °C	61
1%			75
1.25%			100
1.5%			125

**Table 2:** Statistics of friction coefficient and wear efficiency.

Roughness of Samples	Friction Coefficient with 500 g Load—Water Wetting	Wear Efficiency of Zirconia Grinding Balls
61 nm	0.3441	20%
75 nm	0.3077	12.2%
100 nm	0.2883	9.9%
125 nm	0.3356	17.3%

## Data Availability

The original contributions presented in this study are included in the article. Further inquiries can be directed to the corresponding authors.

## Abbreviations

The following abbreviations are used in this manuscript:

CMP	chemico-mechanical polishing
